# NLRP3 Inflammasome and Pathobiology in AMD

**DOI:** 10.3390/jcm4010172

**Published:** 2015-01-14

**Authors:** Lucia Celkova, Sarah L. Doyle, Matthew Campbell

**Affiliations:** 1 Neurovascular Genetics Laboratory, Smurfit Institute of Genetics, Trinity College Dublin, Lincoln Place Gate, Dublin 2, Ireland; E-Mail: celkoval@tcd.ie; 2 Department of Clinical Medicine, School of Medicine, Trinity College Dublin, Dublin 2, Ireland; E-Mail: doyles8@tcd.ie

**Keywords:** age-related macular degeneration, NLRP3 inflammasome, IL-18, geographic atrophy, choroidal neovascularization, retinal pigment epithelium, pyroptosis, autophagy

## Abstract

Age-related macular degeneration (AMD) is the leading cause of central vision loss and blindness in the elderly. It is characterized by a progressive loss of photoreceptors in the macula due to damage to the retinal pigment epithelium (RPE). Clinically, it is manifested by drusen deposition between the RPE and underlying choroid and accumulation of lipofuscin in the RPE. End-stage disease is characterized by geographic atrophy (dry AMD) or choroidal neovascularization (wet AMD). The NLRP3 inflammasome has recently been implicated in the disease pathology. Here we review the current knowledge on the involvement of this multiprotein complex and its effector cytokines interleukin-1β (IL-1β) and IL-18 in AMD progression. We also describe cell death mechanisms that have been proposed to underlie RPE degeneration in AMD and discuss the role of autophagy in the regulation of disease progression.

## 1. Introduction

Age-related macular degeneration (AMD) is a progressive retinal disorder characterized by the loss of central vision due to damage to the macula. Although the exact causes of AMD are still not fully understood, a number of genetic and environmental risk factors have been associated with the disease. A recent meta-analysis of genome-wide association studies identified 19 genetic loci associated with increased susceptibility to AMD, including genes involved in the regulation of complement activity, lipid metabolism, extracellular matrix remodeling and angiogenesis [1]. Additional factors, such as advanced age, gender, race, smoking and oxidative stress, are well established within the field that can lead to a substantially increased risk of developing AMD. Factors such as prolonged exposure to sunlight, obesity, hypertension, diet, especially high dietary intake of vegetable fat and cholesterol and low dietary intake of antioxidants, zinc and vitamin D may also play a role [2,3,4,5,6,7,8]. In recent years, the disease has also been associated with a state of chronic local low-grade inflammation of the retina accompanied by the activation of resident immune cells, production of various inflammatory mediators and infiltration of peripheral immune cells to the site of retinal damage [9,10].

The main clinical hallmark and one of the early signs of AMD development is deposition of drusen between the retinal pigment epithelium (RPE) and the underlying choroid. The major constituents of drusen include, for example, glycoprotein vitronectin, serum amyloid P component, apolipoprotein E, immunoglobulin light chains, coagulation factor X (thrombokinase), complement proteins, serum albumin, Alzheimer’s Aβ-peptide, tissue inhibitor of metalloproteinases 3 (TIMP3) and clusterin (apolipoprotein J) [11,12]. Interestingly, these components are not exclusive to drusen found in AMD, but are common to extracellular deposits associated with other diseases, such as atherosclerosis, elastosis, amyloidosis and dense deposit disease [11]. In addition, at least 40% of drusen are composed of lipids, mostly esterified cholesterol and phosphatidylcholine [13]. In line with the hypothesis that oxidative stress contributes to AMD pathology, products of lipid oxidation (such as carboxyethyl pyrrole (CEP)-protein adducts) and oxidative protein modifications, including abnormal protein cross-links and advanced glycation end products, are also highly abundant in AMD drusen [14]. Another major hallmark of AMD is the accumulation of lipofuscin in RPE cells. Lipofuscin is a complex aggregate of pigmented material that is a result of incomplete lysosomal degradation of phagocytosed photoreceptor outer segments. The precise composition of lipofuscin is not clear, however, one of its major known constituents is a fluorophore A2E, which is a byproduct of the visual cycle [15].

Advanced (late) AMD occurs in two clinical forms. Non-neovascular, also known as non-exudative or “dry”, AMD is characterized by geographic atrophy (GA) extending to the center of the macula, involving the loss of RPE and photoreceptors due to cell death. On the other hand, neovascular AMD, also called exudative or “wet” AMD, is characterized by choroidal neovascularization (CNV), involving abnormal growth of choroidal blood vessels through the Bruch’s membrane/RPE. Unlike normal blood vessels, these CNVs are permeable to blood components resulting in leakage into the sub-retinal space. Therefore, additional manifestations of this form of AMD might also include subretinal fluid accumulation, lipid deposition, hemorrhage, RPE detachment and inevitably fibrotic scarring. Although this form is less common (approx. 10%–15% of AMD patients), it is much more severe and leads to acute (rapid) vision loss [6].

According to the World Health Organization (WHO), 285 million people worldwide (approx. 4% of world’s population) are affected by visual impairment, 14% of them being blind and 86% having low vision. More importantly, 65% of all visually impaired and 82% of all blind people are 50 years or older [16]. These data, however, only include patients suffering from moderate or severe visual impairment.

AMD is the primary cause of visual impairment in industrialized countries [16,17]. A recent systematic review and meta-analysis estimated the global prevalence of AMD (including its very early stages) to be 8.7% in people over the age of 45. Prevalence consistently increased with age in all ethnic groups and geographic regions and globally, approximately 1 in 5 people in the age group 70–79 suffered from some form of AMD. The risk was even more pronounced in the age group 80–84 with every fourth person being an AMD patient [18].

In the United States, AMD is the leading cause of vision loss in people 60 years or older. As many as 11 million Americans suffer from some form of AMD, a number similar to that of people diagnosed with all types of invasive cancer. Moreover, the prevalence of the disease is expected to double by 2050. In fact, more than 2 million people in the Unites States are currently living with the most advanced forms of AMD [19].

The situation is even more critical in Europe, where 18.25% of people over 45 suffer from some form of AMD. AMD prevalence reaches 32.52% in the age group 70–79 and this increases to 45.33% in people 80–84 years of age, meaning that almost every second person in this age group has a chance of developing the disease. It is projected that by 2020 almost 59 million Europeans will develop some form of AMD, this number increasing to almost 70 million in 2040 [18,20,21].

## 2. The NLRP3 Inflammasome and Its Role in AMD

Inflammasomes are large multiprotein complexes that function in the innate immune response as molecular platforms for activation of caspase-1 and subsequent maturation and secretion of biologically active interleukin-1β (IL-1β) and IL-18 [22,23]. NLRP3 (also known as NALP3 or cryopyrin) is a member of the NOD-like receptor (NLR) family. NLRs are cytosolic pattern recognition receptors (PRRs) that share a common structure, comprising three structural domains. A central nucleotide-binding domain (NACHT) mediates ATP-dependent self-oligomerization. It is flanked by *C*-terminal leucine-rich repeats (LRRs) that sense the presence of ligand and a variable *N*-terminal interaction domain responsible for homotypic protein-protein interactions. This *N*-terminal domain can be either a caspase recruitment domain (CARD), pyrin domain (PYD), acidic transactivating domain or baculovirus inhibitor repeat. PRRs recognize common motifs that are broadly shared by groups of related pathogens (pathogen-associated molecular patterns, PAMPs), thereby initiating an inflammatory response to clear infection and restore homeostasis. They also detect products of stressed, injured or dying cells (danger-associated molecular patterns, DAMPs), which then leads to sterile inflammation to facilitate tissue repair. PRRs are linked to intracellular signal transduction pathways that activate various cellular responses mainly involved in promoting inflammation, cytoprotection or tissue repair, often through the activation of NF-κB, AP-1 or IRF transcription factors. NLRP3 can assemble into a large oligomeric structure, by recruitment of an adaptor protein ASC (PYCARD) and procaspase-1, which interacts through its CARD domain with the CARD domain of ASC. In this way, the NLRP3 inflammasome provides a scaffold for autocatalytic cleavage of inactive procaspase-1 into its mature and active p10/p20 tetramer.

The NLRP3 inflammasome is activated in response to a variety of stimuli, including both infectious stimuli and sterile danger signals caused by cellular stress, such as a high concentration of extracellular ATP and hyaluronan released from dying cells, β-amyloid fibrils associated with Alzheimer’s disease, increased extracellular glucose, crystals of monosodium urate or cholesterol, a decrease in extracellular osmolarity or pH, extracellular matrix components, aluminium vaccine adjuvants, environmental and industrial particles, such as silica and asbestos, or nanoparticles [24,25,26,27,28,29,30,31,32,33,34,35,36]. It has been suggested that all PAMPs and DAMPs might activate the inflammasome through a common general pathway. Although this mechanism has not yet been identified, several different models have been proposed, including increased K^+^ efflux, Ca^2+^ signaling, damage to the phagolysosomal membrane and the activity of protein-degrading enzymes cathepsins, the generation of reactive oxygen species (ROS) and the release of oxidized DNA from mitochondria, TAK1 activation or NLRP3 deubiquitination [26,27,30,34,35,37,38,39,40,41,42,43,44]. This also suggests that NLRP3 is more a sensor of cellular stress rather than a true receptor. According to the current literature, activation of the inflammasome and subsequent production of mature IL-1β and IL-18 is a two-step process, requiring two signals. Classically, the first step, referred to as inflammasome priming, involves NF-κB-mediated synthesis of the inactive precursors pro-IL-1β and pro-IL-18, in response to recognition of a specific ligand by its corresponding PRR, and up-regulation of inflammasome components, including NLRP3 [45]. A second signal is required for NLRP3 oligomerization, recruitment of ASC and procaspase-1 and subsequent cleavage of procaspase-1 into its active form, leading to processing of pro-IL-1β and pro-IL-18 and eventually release of mature cytokines IL-1β and IL-18 [23].

The NLRP3 inflammasome was first implicated in AMD in 2012 by four independent studies, including one from our own laboratory [46,47,48,49]. We showed that drusen isolated from donor AMD eyes as well as drusen component C1Q (a complement protein) could activate the NLRP3 inflammasome and caspase-1 in peripheral myeloid and mononuclear cells, leading to secretion of IL-1β and IL-18. Interestingly, NLRP3 inflammasome activation by C1Q was dependent on phagolysosome activity and cathepsin B. We also detected NLRP3 and cleaved caspase-1 in activated macrophages in the retina after immunization of mice with CEP-adducted mouse serum albumin to model a dry AMD-like pathology, however, we did not determine whether this contributed to the pathology observed in this mouse model. In a commonly used murine model of neovascular AMD, NLRP3 was shown to have a protective role as laser-induced CNVs were exacerbated in *Nlrp3^−/−^* mice [46]. Another group showed that the NLRP3 inflammasome was present in RPE and in adjacent drusen during pathogenesis of both dry and wet AMD. Consistent with our observations, they also demonstrated that activation of the inflammasome and caspase-1 in RPE cells, with the subsequent release of mature IL-1β, occurred by lysosomal destabilization and release of cathepsins [47]. Moreover, *NLRP3* mRNA was up-regulated in response to oxidative damage [48].

It has been shown that the NLRP3 inflammasome was activated in GA in response to repetitive transposable elements of non-coding RNA, termed *Alu* RNA, that accumulated in RPE due to a loss of a miRNA processing enzyme DICER1. The *Alu* RNA-mediated RPE cytotoxicity occurred independently of TLRs or other canonical RNA sensors, but was dependent on the adaptor protein MyD88. Moreover, *Alu* RNA was suggested to function as both a priming agent and activator of the inflammasome. Up-regulation of *NLRP3* and *IL-18* mRNA in response to *Alu* RNA occurred through the production of ROS by mitochondria independently of MyD88. *Alu* RNA was also required for the oligomerization of NLRP3 and recruitment of ASC to subsequently activate caspase-1 in the inflammasome complex. Interestingly, MyD88 was suggested to function downstream of the inflammasome as MyD88 inhibition did not prevent *Alu* RNA-induced caspase-1 activation, whereas caspase-1 inhibition reduced *Alu* RNA-induced phosphorylation of IRAK1/4, molecules downstream of MyD88. Clinically, the RPE of human subjects suffering from GA show a high abundance of *NLRP3*, *IL-18* and *IL-1β* mRNA compared to normal healthy eyes, which was accompanied by an increase at the protein level of NLRP3, ASC, caspase-1 p20 and phosphorylated forms of IRAK1 and IRAK4. The authors of this study therefore proposed that DICER1 was an important negative regulator of NLRP3 inflammasome activation in RPE and that genetic or pharmacological inhibition of inflammasome components or MyD88 might prevent RPE degeneration due to DICER1 loss or *Alu* RNA exposure in dry AMD [49].

Later studies confirmed that other drusen components, such as the Aβ-peptide 1–40, could increase expression of inflammatory mediators and inflammasome components in the retina and RPE *in vivo*, including *IL-6*, *TNF-α*, *IL-1β*, *IL-18*, *NLRP3* and *caspase-1* mRNAs. Intravitreal injection of Aβ-peptide 1–40 was also responsible for elevated levels of mature IL-1β and IL-18 in the vitreous [50]. Lipofuscin component A2E was shown to induce a consistent and robust production of cytokines and chemokines in the RPE cells, including IL-1β, which was mediated by NLRP3 inflammasome activation through A2E endocytosis and cathepsin activity. Consistent with these findings, increased levels of IL-1β were observed in the RPE of *ABCA4^−/−^* mice, which demonstrated elevated lipofuscin and A2E levels in the RPE with ageing [51]. Importantly, an increase in vascular endothelial growth factor A (VEGF-A), associated with neovascularization, also resulted in NLRP3 inflammasome activation in RPE cells in a transgenic mouse model of AMD, mediated by oxidative damage. Interestingly, NLRP3 deficiency in this mouse model reduced the number of VEGF-A-induced CNV lesions and RPE barrier breakdown, suggesting involvement of the inflammasome in the pathology of both forms of AMD [52].

## 3. Effects of IL-1β and IL-18 on RPE in AMD

IL-1β and IL-18 are both members of the IL-1 cytokine family. As discussed above, they are synthesized as inactive precursors pro-IL-1β and pro-IL-18 and require cleavage by caspase-1 to produce mature, biologically active cytokines. Interestingly, pro-IL-18 was shown to be constitutively expressed in a large variety of cells, whereas pro-IL-1β expression had to be induced through NF-κB-mediated activation of the *IL-1β* promoter [53,54]. IL-1β is considered a classic activator and mediator of inflammation, its deregulation has been implicated in a range of autoimmune diseases and strategies abolishing IL-1β signaling have proven efficient in treatment of many of these conditions [55,56,57]. Interestingly, IL-1β also functions as a potent pro-angiogenic factor by stimulating the production of VEGF [58,59,60,61,62,63]. The role of IL-18, on the other hand, appears to be much more complex. It can stimulate both Th1 and Th2 responses depending on the local microenvironment [64,65] and its activity is balanced by a naturally occurring high affinity IL-18-binding protein (IL-18BP). A role for IL-18 has been implicated in a number of inflammatory conditions, whereas other disease models have shown protective effects of IL-18 [66,67]. This highlights a dichotomous role for IL-18 that can play either a protective and/or a pro-inflammatory role, depending on the immunological status of the cell or the type and phase of the inflammatory process, as suggested by Bamias *et al.* [68]. The discovery of NLRP3 activation in AMD (discussed above) has prompted investigations into the role of IL-1β and IL-18 in AMD pathobiology.

Early studies showed that IL-1β could induce expression and secretion of the chemokines IL-8 and MCP-1 in RPE cells, mediated by activation of NF-κB and MAPK pathways, including co-activation of p38 and ERK1/2 [69]. *In vitro* experiments revealed that chronic IL-1β treatment over an extended period of time increased permeability of the RPE monolayer and affected expression of tight junction proteins [70], whereas a short treatment with mature IL-1β did not have any significant effects on RPE [71]. Moreover, even a long-term treatment of RPE cells with IL-1β did not have any significant effects on cell viability and did not induce apoptosis *in vitro* [70]. Indeed, it may be more likely that in the context of AMD, the immune cells (microglia and macrophages) contribute more to the production of IL1-β than the RPE. Activation of the NLRP3 inflammasome in models of AMD suggested that IL-1β might be responsible or at least contribute to disease pathology [47]. We have shown that *Il1r1* gene knockout did not have any significant effects on laser-induced CNV development in mice [46]. Others, however, have demonstrated that *Il1r1* deficiency, abolishing IL-1β signaling, reduced the number of CNV lesions in a transgenic mouse model of AMD characterized by increased levels of VEGF-A in the RPE, retina and serum [52]. Similarly, administration of the IL-1 receptor antagonist anakinra decreased the area of laser-induced CNVs in a rat model [72].

Our initial studies proposed that NLRP3 conferred protection against CNV development through IL-18, since *Il18^−/−^* mice (similar to *Nlrp3^−/−^*) showed exacerbated laser-induced CNVs, directly implicating IL-18 in CNV progression [46]. These findings were confirmed by others who observed a significant increase in the number of CNV lesions in a transgenic mouse model bred on an *Il18^−/−^* background [52]. A recent study by our laboratory demonstrated that exogenous administration of mature recombinant IL-18, both intravitreal and systemic, attenuated laser-induced CNV development in mice. We also showed that IL-18 had no effect on RPE cell viability or RPE barrier integrity, while remaining biologically active and stimulating NF-κB and p38 MAPK pathways [73]. In addition, we have also recently shown that clinical grade IL-18 has no effect on retinal or RPE integrity when injected intra-vitreally in cynomolgus monkeys even at doses as high as 10,000 ng [74]. IL-18 was also revealed by other groups to function as an anti-angiogenic and anti-permeability factor by regulating VEGF production and counteracting VEGF-induced RPE barrier breakdown [46,73,75]. Contrary to these findings, *Alu* RNA accumulation due to DICER1 deficiency was shown to increase secretion of IL-18, but not IL-1β, in human RPE cells after NLRP3 inflammasome activation, suggesting that IL-18 was the effector molecule in *Alu* RNA-mediated RPE cytotoxicity. It was also observed that neutralizing IL-18, but not IL-1β, reversed *Alu* RNA-induced RPE degeneration. Moreover, an initially un-disclosed dose of recombinant IL-18 was found to induce RPE cell death independently of caspase-1. Emanating from these observations, together with the ones regarding NLRP3 inflammasome activation (discussed above), a model was proposed whereby IL-18 produced by an activated inflammasome in RPE cells in response to *Alu* RNA acts on the same cell type to activate MyD88 through the IL-18 receptor and could cause RPE degeneration via an IRAK1/4-mediated mechanism [49].

## 4. RPE Cell Death in AMD

As discussed above, exposure of RPE cells to the cytokines IL-1β and IL-18 *in vitro* did not induce RPE cell death [70,73]. Moreover, intravitreal or systemic administration of physiologically relevant doses of mature recombinant IL-18 was not associated with any pathological effects on mouse RPE cells *in vivo* [73,75]. It is likely that these cytokines are not directly responsible for RPE cell death seen in geographic atrophy.

It is more probable that NLRP3 inflammasome activation through lysosomal destabilization and cathepsin release induce significant RPE cytotoxicity, independent of IL-1β and IL-18 secretion. Moreover, this potential mechanism has been shown to be dependent on caspase-1 activity, indicating that an inflammasome-mediated cell death mechanism might contribute to AMD-associated GA [47]. Pyroptosis is a type of cell death which is inherently dependent on caspase-1. It has evolved as an innate immune effector mechanism against intracellular bacteria [76]. Pyroptosis is characterized by pore formation in the plasma membrane, which disrupts cellular ionic gradients, leading to increased osmotic pressure, water influx, cell swelling and subsequent plasma membrane rupture and release of pro-inflammatory intracellular contents. DNA cleavage and nuclear condensation also occur during pyroptosis, however, these are distinct from DNA laddering seen in apoptosis since nuclear integrity is preserved (Table 1). Caspase-1 also cleaves and inactivates metabolic enzymes, which might limit cellular energy supply during pyroptosis [22,77,78]. As pyroptosis is dependent on caspase-1, it is obvious that prolonged activation of NLRP3 inflammasome could potentially induce pyroptotic cell death. Indeed, we have observed that over-expression of pro-IL-1β and pro-IL-18 caused cell swelling, which might be an indication of pyroptosis [73].

**Table 1:** Cardinal features of major types of cell death.

	Characteristics	Apoptosis	Pyroptosis	Necrosis
Morphology	Cell lysis	NO	YES	YES
	Cell swelling	NO	YES	YES
	Pore formation	NO	YES	YES
	Membrane blebbing	YES	NO	NO
	DNA fragmentation	YES	YES	YES
Mechanism	Caspase-1	NO	YES	NO
	Caspase-3	YES	NO	NO
	Cytochrome-c release	YES	NO	NO
Outcome	Inflammation	NO	YES	YES
	Programmed cell death	YES	YES	NO

Recent evidence has also revealed that some necrotic cell death in the RPE may be regulated by RIP3, which is called “necroptosis” or programmed necrosis [79,80,81]. Indeed, in GA, necrotic cell death mechanisms may be the fundamental part of the cell death modality that contributes to the observed pathology in human subjects. For example, this may not be only limited to the RPE as necrotic features may have been observed in the work of Shelley *et al.* [82] that reported dying cones displaying swelling and enlargement consistent with necrosis.

Others proposed that RPE cell death associated with GA might be mediated by a mechanism not relying on caspase-1. Lipofuscin component A2E could cause RPE cell loss independently of caspase-1, but the process was dependent on A2E internalization [51]. *Alu* RNA-induced RPE degeneration was proposed to occur via a mechanism other than pyroptosis, as it was observed that the cytoprotective agent glycine (a known inhibitor of pyroptosis) did not prevent *Alu* RNA-mediated RPE cell death. Moreover, IL-18 as the key effector of *Alu* RNA-induced cytotoxicity in the proposed model, could cause RPE degeneration in *Casp1^−/−^* mice [49]. In fact, it was postulated that *Alu* RNA accumulation, resulting from DICER1 deficiency, exerts its cytotoxic effect on RPE cells through caspase-3-mediated apoptosis [83]. However, another study showed that there was no evidence of apoptosis in response to a drusen component Aβ-peptide 1–40 [50].

**Figure 1:**
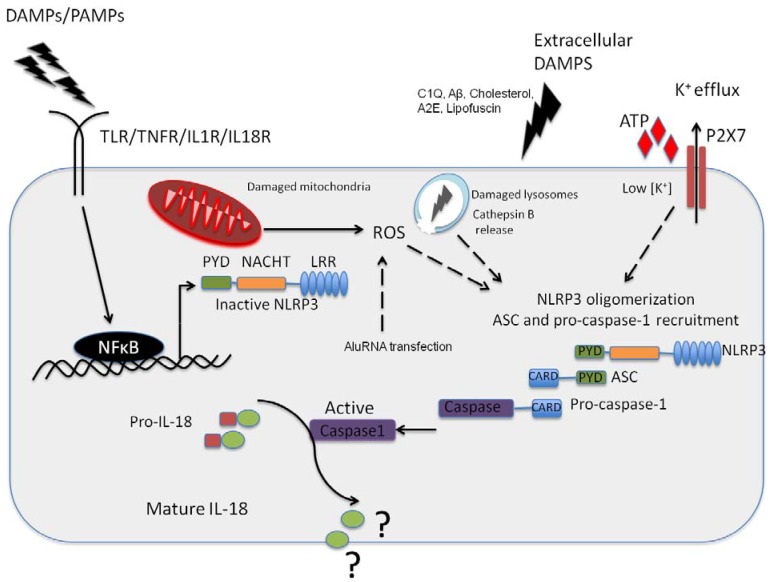
NLRP3 inflammasome activation in the retinal pigment epithelium (RPE). Pathogenic Pathogen Associated Molecular Patterns (PAMPs) from virus or bacteria, or sterile Danger or Damage Associated Molecular Patterns (DAMPs) can activate NFkappaB leading to the expression of NLRP3 NLRP3 can then oligomerise, recruiting ASC and pro-caspase-1 in response to an activation signal such as ATP, C1Q, amyloid-β, A2E or Lipofuscin in the context of age-related macular degeneration (AMD). NLRP3 can also be activated in response to K^+^ efflux through the ATP gated P2X7 channel, which is a response to reactive oxygen species acc8umulation in the RPE. In addition, this can be in in resopnse to cathepsin B release from damaged lysosomes. It has yet to be fully resolved as to the function of IL-18 in the RPE, but this cytokine appears to be constitutively expressed in the RPE.

Apoptosis is a form of programmed cell death that is mechanistically defined by the requirement for particular caspases (such as caspase 2, 3, 6, 7, 8, 9, and 10) which mediate an orchestrated disassembly of the cell. Fundamentally characteristic features of apoptosis include cytoplasmic and nuclear condensation and DNA cleavage distinct from the ones associated with pyroptosis, maintenance of an intact plasma membrane, loss of mitochondrial integrity, release of cytochrome-c and a distinct absence of inflammation (see Table 1) [77]. Overall, the role of the inflammasome as it pertains to RPE health and disease is still yet to be fully resolved but is very briefly summarized in Figure 1.

## 5. Role of Autophagy in Inflammasome Regulation in AMD

Autophagy can be defined as “a programme of cellular self-digestion in which cytoplasmic components are sequestered and degraded intracellularly in autophagosomes” [77]. The process starts with the formation of an isolation membrane (phagophore) which eventually fuses with itself to enclose its target in a double-membraned autophagosome. The autophagosome then migrates through the cytoplasm to fuse with a lysosome, leading to degradation of autophagolysosomal content. Autophagy is an important regulator of energy and nutrient homeostasis, plays an essential role in tissue development and affects immune responses. Most importantly, autophagy regulates endogenous inflammasome activators as well as expression of inflammasome components and pro-IL-1β, thus being a key modulator of IL-1β and IL-18 transcription, processing and release [84,85]. Studies have demonstrated that inhibition of autophagy leads to accumulation of damaged mitochondria producing ROS, which in turn causes NLRP3 inflammasome activation [86,87]. Autophagy has also been shown to control IL-1β secretion by targeting pro-IL-1β for lysosomal degradation and by regulating NLRP3 inflammasome activation. Stimulating autophagy *in vivo* decreased serum levels of IL-1β in response to LPS challenge [88]. Interestingly, activation of the inflammasome was also found to induce autophagy. Moreover, blocking autophagy increased inflammasome activation, whereas induction of autophagy limited it, suggesting that autophagy might be a negative regulator of inflammasome activity. Assembled inflammasomes were ubiquitinated, which targeted them for delivery to the autophagosome, subsequently leading to their destruction. This demonstrated that autophagy could function as a mechanism to limit excessive inflammation by directly eliminating active inflammasome complexes [89]. In line with these findings, autophagy was shown to protect against pyroptosis. It was observed that when autophagy was suppressed, pyroptosis occurred more frequently possibly because of sustained inflammasome activation [90].

These observations, together with the recent association of the NLRP3 inflammasome with AMD pathology, indicate that autophagy might play a fundamental role in AMD progression. Indeed, the RPE cell swelling that was observed after over-expression of pro-IL-1β and pro-IL-18, was abrogated by inducing autophagy, possibly due to sequestration and subsequent degradation of excessive cytokine precursors in the autophagosome [73]. Stimulating autophagy could decrease IL-1β-induced VEGF levels, which may have important implications in CNV development associated with wet AMD [63]. Interestingly, autophagy markers were also present in AMD drusen and an increase in these markers was observed with increased age. Stimulating autophagy in RPE cells, by inducing damage to mitochondrial DNA, did not alter phagocytic activity of the RPE, however, it significantly impaired lysosomal function through a decrease in cathepsin activity. An increase in exocytosis was also observed and exosomes were shown to contain proteins found in human drusen [91]. In addition, a non-canonical form of autophagy has also been shown to promote the visual cycle by supporting degradation of photoreceptor outer segments and maintaining retinoid levels within the RPE [92].

## 6. Discussion

AMD remains a significant public burden in many countries worldwide, especially in the western world. The only effective AMD treatment currently involves intravitreal injections of anti-VEGF drugs that target CNV development in neovascular (wet) AMD [93]. At present, there is no treatment for dry AMD-associated GA. To some extent, the disease management can be achieved through recommended dietary supplementation and lifestyle changes. Clearly, a deeper understanding of the pathological mechanisms that underlie AMD development and progression is necessary to provide new strategies for more efficient therapies. However, this would also require better *in vitro* and *in vivo* models than those currently available, that would reliably reflect AMD-associated pathologies.

The most important clinical hallmarks of early AMD are drusen deposition and lipofuscin accumulation. It has been shown that a number of drusen components as well as A2E fluorophore, the major constituent of lipofuscin, could activate NLRP3 inflammasome which serves as a molecular platform for activation of caspase-1 and subsequent processing and activation of IL-1β and IL-18 [46,50,51]. Similarly, oxidative stress which increases the risk of AMD development, has also been associated with inflammasome activation [41,42,48,49,52]. Interestingly, the NLRP3 inflammasome in the context of AMD is not only activated in innate immune cells [46], but also in the RPE itself [47,48,49,50,51,52].

It was suggested that the NLRP3 inflammasome might be protective against CNV development and this protection was mediated by IL-18 through its anti-angiogenic effects [46]. However, He and Marneros argued that the laser-induced CNV model that was used in the study is more a model of an acute wound healing process, as these CNVs are induced in the context of healthy eyes, and thus represents a very limited model of neovascular AMD [94]. Using a VEGF-based transgenic mouse model it was observed that NLRP3 inflammasome activation and secretion of its effector cytokine IL-1β promoted AMD-associated pathologies, including RPE barrier breakdown and CNV lesion formation [52], possibly through the strong angiogenic properties of IL-1β [58,59,60,61,62,63]. This was supported by the fact that administration of the IL-1 receptor antagonist anakinra has previously been shown to decrease the area of laser-induced CNVs in an animal model of AMD [72]. Nonetheless, these studies were consistent in the fact that IL-18 deficiency promoted CNV development, suggesting an important protective role of IL-18 in neovascular AMD pathology. This was also confirmed by exogenous administration, intravitreal and systemic, of mature recombinant IL-18 into a mouse model of wet AMD [73]. In line with these findings, it was previously suggested that IL-18 might be protective in other diseases, such as experimental colitis and colorectal cancer in mice [95,96,97,98]. Moreover, it has been shown to function as an essential regulator of angiogenesis, not only in the retina [99,100,101,102].

While inflammasome priming is generally mediated by NF-κB and the production of ROS in mitochondria, *Alu* RNA-mediated inflammasome oligomerization and assembly was reported to be dependent on the purinergic receptor P2X7, a ligand-gated ion channel which opens in response to extracellular ATP binding and causes K^+^ efflux [103]. Other studies, however, support the two-signal model of NLRP3 inflammasome activation and showed that priming signals increased NF-κB-mediated expression of pro-IL-1β, pro-IL-18 and NLRP3 and the inflammasome assembly was subsequently induced by lysosomal destabilization [46,47].

Similar to other cell types, RPE cells do not constitutively express pro-IL-1β. NF-κB-mediated activation of the *IL-1β* promoter is required to induce its synthesis and subsequent activation of inflammasome is necessary to process this inactive precursor into its mature form [47,54]. However, RPE cells constitutively express pro-IL-18 and also basal levels of inflammasome components, including NLRP3, ASC and procaspase-1 [46,47]. This expression might be regulated via TLR4 which was shown to participate in the transmembrane signaling during phagocytosis of photoreceptor outer segments by the RPE [104].

Currently, the mechanism of RPE cell death associated with GA in dry AMD is far from clear. Several reports indicated that this process might be dependent on caspase-1 and the inflammasome, directly implicating pyroptosis as the main pathway of RPE cell loss [47]. Interestingly, only one NLRP3 inflammasome complex is formed in a cell at a time [105]. However, the size of this complex has been reported to be 2 μm in diameter [106], as inflammasomes have been shown to assemble into large aggregates of prion-like fibers [106,107]. RPE cells within the macular region are tall (14–16 μm) and narrow (10–14 μm) [108]. With advanced age, the height of macular RPE cells increases, which might be an indication of cell swelling. Comparing the size of an inflammasome with the size of an RPE cell, it is clear that, when formed, the inflammasome complex takes up a substantial proportion of the cell cytoplasm. Therefore, the size of the inflammasome itself together with activation of caspase-1 could result in progressive deregulation of cell volume with subsequent rupture of the plasma membrane and release of the intracellular content, which are features classically associated with pyroptosis. However, it has been proposed that GA-associated RPE cell degeneration cannot occur through pyroptosis but is facilitated by caspase-3-dependent apoptosis which is triggered via IL-18 receptor signaling mediated by MyD88 [49,83]. If this is the case, it would represent a paradigm shift in the field of apoptosis. MyD88 is a key adaptor molecule downstream of several inflammatory receptors. MyD88-associated signaling pathways always converge to activate NF-κB, AP-1 or IRF transcription factors, which are classical pro-inflammatory mechanisms that enhance inflammatory responses [109]. Apoptosis, on the other hand, is fundamentally an anti-inflammatory type of cell death with its main function being to limit the inflammatory response. The direct association between these two apparently opposing pathways has never been described before. Also, owing to the size of the inflammasome complex, a large energy supply would be required to degrade such a big aggregate in an anti-inflammatory fashion through apoptosis. On the other hand, pyroptosis limits energy supply of the cell through caspase-1 and could represent an efficient way of eliminating a deregulated and hazardous inflammasome due to its prolonged activation. However, a recent study has described that nucleoside reverse transcriptase inhibitors (NRTIs) in mouse models of geographic atrophy and choroidal neovascularization can abrogate disease pathology in a manner dependent on inhibiting the inflammasome. This suggests that NRTIs could be used as a therapeutic tool for both forms of AMD. These studies were conducted in mouse models of the condition and time will tell if they translate to clinically beneficial results in human subjects [110].

The NLRP3 inflammasome is now known to be tightly regulated by autophagy and a role for autophagy is now also recognized in AMD pathology. Autophagy can target pro-IL-1β for lysosomal degradation, eliminates assembled inflammasome complexes and blocks pyroptosis to limit the inflammatory response [88,89,90]. It is now well accepted that autophagy function decreases with ageing. Although an increase in autophagic markers is associated with drusen and the RPE of older animal models, the process of lysosomal degradation, the final stage of autophagy, was substantially impaired in these animals [91]. Therefore, although there is an apparent increase in autophagy, possibly to facilitate degradation of the increased load of damaged organelles, especially mitochondria, in aged animals, the process of degradation was not completely finished and autophagy failed. This might in turn lead to accumulation of partially degraded intracellular waste products, such as lipofuscin. Alternatively, clearance of these intracellular proteins might be achieved through exocytosis, which leads to their deposition outside the RPE as drusen [91] where they can be recognized and cleared by the immune system. This is in line with observations that macrophages that infiltrate the retina in the early stages of AMD are M2-polarized and are associated with wound healing [94]. However, it can be speculated that excessive tissue damage can create a suitable microenvironment to “switch” these cells into their M1 polarization state associated with promotion of an inflammatory response.

A recently described VEGF-based transgenic mouse model for AMD suggested that increased VEGF alone is sufficient to cause both forms of the disease [52,111]. Although targeting VEGF has proven to be very efficient in the treatment of neovascular AMD [93], this form of treatment is relatively expensive, especially due to the frequency of anti-VEGF injections that are required. More importantly, however, some will eventually become resistant to such treatment. The data discussed above collectively suggest a dual role for NLRP3 in AMD progression. It might be protective against AMD-associated pathologies through IL-18-mediated anti-angiogenic effects but, alternatively, the formation of an inflammasome itself may be detrimental to the RPE. Indeed, some have proposed that genetic or pharmacological suppression of NLRP3 inflammasome components, its effector cytokines or MyD88 might be a successful approach to AMD treatment [49,112]. An ideal strategy to treat both forms of AMD could potentially involve the use of current anti-VEGF therapy, together with inhibition of IL-1β and administration of recombinant IL-18 or potentiating the release of naturally occurring IL-18, possibly through an interference with IL-18BP. This is also supported by our recent observation that anti-VEGF therapy works more effectively in combination with IL-18 [73].

Interestingly, although IL-18 is a naturally occurring molecule, the endogenously produced IL-18 alone does not have the capacity to prevent wet AMD development in humans. This might be due to the presence of IL-18BP, which can sequester IL-18. In addition, as RPE degeneration progresses, infiltrating macrophages can polarize, which in turn can activate retinal glial cells to produce pro-angiogenic IL-1β. As such, IL-18 that is also produced does not have the capacity to balance the resulting and overwhelming load of VEGF. In addition, this model could also explain why some patients with dry AMD would progress to develop the more severe neovascular form of the disease.

## 7. Conclusions

- The NLRP3 inflammasome appears to play a central role in both atrophic and neovascular AMD.
- Mature IL-18 and IL-1β can be produced by both the RPE and systemic/resident myeloid derived cells, however whether mature cytokines can induce RPE specific apoptosis is still unclear.
- A robust elucidation of the role played by the NLRP3 inflammasome in atrophic and neovascular AMD may lead to potential strategies to treat both clinical forms of the condition.
